# Robotic vs laparoscopic distal gastrectomy with D2 lymphadenectomy for gastric cancer: a retrospective comparative mono-institutional study

**DOI:** 10.1186/s12893-016-0180-z

**Published:** 2016-09-20

**Authors:** Fabio Cianchi, Giampiero Indennitate, Giacomo Trallori, Manuela Ortolani, Beatrice Paoli, Giuseppe Macrì, Gabriele Lami, Beatrice Mallardi, Benedetta Badii, Fabio Staderini, Etleva Qirici, Antonio Taddei, Maria Novella Ringressi, Luca Messerini, Luca Novelli, Siro Bagnoli, Andrea Bonanomi, Caterina Foppa, Ileana Skalamera, Giulia Fiorenza, Giuliano Perigli

**Affiliations:** 1 Department of Surgery and Translational Medicine, Center of Oncological Minimally Invasive Surgery (COMIS), University of Florence, Largo Brambilla 3, 50134 Florence, Italy; 2 IFCA, Florence, Italy; 3 Unit of Gastroenterology, University Hospital Careggi, Florence, Italy; 4 ISPO, Florence, Italy; 5 Department of Experimental and Clinical Medicine, University of Florence, Florence, Italy

**Keywords:** Gastric cancer, Robotic surgery, Laparoscopy, Lymphadenectomy, Distal gastrectomy

## Abstract

**Background:**

Robotic surgery has been developed with the aim of improving surgical quality and overcoming the limitations of conventional laparoscopy in the performance of complex mini-invasive procedures. The present study was designed to compare robotic and laparoscopic distal gastrectomy in the treatment of gastric cancer.

**Methods:**

Between June 2008 and September 2015, 41 laparoscopic and 30 robotic distal gastrectomies were performed by a single surgeon at the same institution. Clinicopathological characteristics of the patients, surgical performance, postoperative morbidity/mortality and pathologic data were prospectively collected and compared between the laparoscopic and robotic groups by the Chi-square test and the Mann-Whitney test, as indicated.

**Results:**

There were no significant differences in patient characteristics between the two groups. Mean tumor size was larger in the laparoscopic than in the robotic patients (5.3 ± 0.5 cm and 3.0 ± 0.4 cm, respectively; *P* = 0.02). However, tumor stage distribution was similar between the two groups. The mean number of dissected lymph nodes was higher in the robotic than in the laparoscopic patients (39.1 ± 3.7 and 30.5 ± 2.0, respectively; *P* = 0.02). The mean operative time was 262.6 ± 8.6 min in the laparoscopic group and 312.6 ± 15.7 min in the robotic group (*P* < 0.001). The incidences of surgery-related and surgery-unrelated complications were similar in the laparoscopic and in the robotic patients. There were no significant differences in short-term clinical outcomes between the two groups.

**Conclusions:**

Within the limitation of a small-sized, non-randomized analysis, our study confirms that robotic distal gastrectomy is a feasible and safe surgical procedure. When compared with conventional laparoscopy, robotic surgery shows evident benefits in the performance of lymphadenectomy with a higher number of retrieved and examined lymph nodes.

## Background

Minimally invasive surgery for gastric cancer has evolved rapidly and has increased in popularity during the last two decades mainly in the Far East and for patients with early-stage tumors [1, 2]. A number of non-randomized trials, randomized controlled trials and meta-analyses have confirmed that laparoscopic surgery for gastric cancer can improve short-term results and the patient’s quality of life when compared with open surgery [3–7]. Nevertheless, the development of laparoscopic surgery for gastric cancers in the Western world has been slow because most gastric cancers are diagnosed in an advanced stage for which laparoscopic gastrectomy is not yet considered an acceptable alternative to standard open surgery [8, 9]. This skepticism is basically due to the technical complexity of laparoscopic gastrectomy and concerns the feasibility of an oncologically acceptable lymphadenectomy. For these reasons, laparoscopic gastrectomy is considered one of the most difficult operations, requiring a long learning curve of about 40–50 cases [10, 11].

Robotic surgery has been introduced to overcome some of the technical limitations of laparoscopic surgery, such as two-dimensional vision, amplified physiological tremor, restricted range of motion and ergonomic discomfort [12, 13]. Robotic systems include operator-controlled 3-dimensional cameras that ensure steady and effective surgical fields of view with motion scaling and multiple degrees of freedom. It is believed that this technological evolution can assist the surgeon with complex surgical procedures that are required in radical gastrectomy, such as precise lymph node dissection and intracorporeal anastomoses [14].

Several studies have compared the feasibility and efficacy of robotic-assisted gastrectomy to that of laparoscopic-assisted gastrectomy for gastric cancer [15]. Robotic gastrectomy was reported to be associated with less operative blood loss and shorter hospital stay than laparoscopic gastrectomy [16, 17]. However, an overt advantage of robotic surgery in comparison with the laparoscopic technique in the treatment of gastric cancer has not been demonstrated yet.

This study was designed to analyze our early experience with robotic gastric surgery and compare the short-term clinical outcomes after laparoscopic and robotic distal gastrectomy for gastric cancer.

## Methods

A total of 41 laparoscopic distal gastrectomies (LDG) for gastric cancer have been performed since June 2008 at the Center of Oncologic Minimally Invasive Surgery (COMIS), University of Florence, Florence, Italy. After the introduction of the daVinci Si surgical system (Intuitive Surgical Inc., Sunnyvale, CA, USA) in April 2014 at our hospital, we have performed 30 robotic distal gastrectomies (RDG) for gastric cancer between June 2014 and September 2015. All of the laparoscopic and robotic procedures were performed by a single surgeon (F.C.) and these cases were his initial experience with robotic gastrectomy.

We prospectively collected and retrospectively compared the clinicopathological characteristics, surgical performance and postoperative outcomes/morbidities between these two groups of patients. All patients underwent diagnostic and preoperative staging work-up according to a standard protocol which includes upper digestive endoscopy with gastric biopsy and computed tomography of the abdomen and chest. Patients with distant metastases, para-aortic lymph node involvement and/or pre- or intraoperative diagnosis of T4 lesions (i.e., local invasion of other organs, including spleen, pancreas or peritoneum), were excluded from the study. All patients had been thoroughly informed about the study and gave their written consent for the investigation in compliance with the Helsinki Declaration and in accordance with the ethical committee of our University Hospital.

The characteristics of patients, such as age, gender, body mass index (BMI) and tumor location, pathological results and surgical outcomes (operative time, blood loss, postoperative morbidity and mortality, time-to-first flatus, time-to-first oral intake and postoperative hospitalization) were collected.

Tumor localization was classified as middle or lower third of the stomach. The extension of lymph node dissection, namely D1 + α/β or D2, was performed according to the lymph node classification of the Japanese Gastric Cancer Association [18]. Tumors were classified according to the 7th edition of the AJCC/TNM tumor staging [19]. They were also classified according to Lauren’s histotype, i.e., intestinal, diffuse or mixed.

### Surgical technique

#### Trocar placement and docking the robotic arms

The preoperative procedures of RDG are not different from those of LDG except for the use of robotic ports and articulating robotic instruments. Under general anesthesia, the patient was placed in supine, reverse Trendelenburg position with legs abducted. In the robotic technique, the camera port was inserted by the open method through an umbilical transverse incision with a 12-mm trocar. After establishing pneumoperitoneum, three 8-mm trocars for the robotic arms were inserted: one in the upper right quadrant, one in the lower right quadrant, and one in the upper left quadrant. A final fourth 12-mm trocar was inserted in the lower left quadrant for the assistant. Either a hook or a monopolar shear was held in the first robotic arm located at the patient’s left side. A Maryland bipolar forceps and a Cadiere forceps were held in the second and third arms, respectively, at the patient’s right side.

The LDG surgical technique includes four trocars (two 12-mm and two 5-mm trocars) that are placed as previously described [20].

#### Distal gastrectomy

Most of the operative steps during RDG were the same as those during LDG. First, a routine exploration of the abdominal cavity was performed. D1 + α/β or D2 lymphadenectomy and gastric dissection were performed as previously described [20]. A key difference between RDG and LDG is that robotic dissection of lymph nodes was performed with the robotic wristed instruments. Moreover, some procedures, such as operating the stapler, applying hemoclips, inserting and removing surgical gauzes, are performed by the first operator during LDG whereas they are performed by the assistant during RDG.

In both procedures, mechanical intracorporeal either Billroth II or Roux-en-Y gastrojejunal anastomosis was performed. In the last 25 laparoscopic and in all robotic procedures, we reinforced the duodenal stump with a running, barbed suture after the duodenal transaction. The surgical specimen was placed in a polyethylene endobag and pulled out of the peritoneal cavity through the umbilical port which was extended to a length of 4–6 cm.

### Statistical analysis

Categorical variables within laparoscopic and robotic groups were compared using Fisher’s exact test or the chi-square test. Quantitative variables were summarized by means and SEM or medians and range. Groups were compared using the Mann-Whitney test.

## Results

Table 1 shows the clinicopathological characteristics of the patients in the LDG and RDG groups. There were no significant differences in terms of age, sex or BMI. Patients in the LDG group had a larger mean tumor size than those in the RDG group. However, tumor stage distribution was similar between the two groups. Most of the tumors were located in the lower third of the stomach in both groups.

**Table 1:** Clinicopathological characteristics of patients undergoing laparoscopic and robotic distal gastrectomy

	Laparoscopic group	Robotic group	*P* value
	*N* = 41	*N* = 30	
Gender (male/female)	19/22	14/16	NS
Age (year) (median, range)	74 (40–87)	73 (45–86)	NS
BMI (kg/m^2^) (median, range)	26.0 (23–30)	27.0 (23–38)	NS
Tumor location			NS
Middle third	17 (41.5 %)	10 (33.3 %)	
Lower third	24 (58.5 %)	20 (66.7 %)	
Lauren classification			NS
Intestinal	19 (46.3)	19 (66.3)	
Diffuse	13 (31.7)	5 (16.7)	
Mixed	9 (22.0)	6 (20.0)	
Tumor size (cm) (mean ± SD)	5.3 ± 0.5	3.0 ± 0.4	*P* = 0.02
Stage distribution			NS
I	15 (36.6)	11 (36.7)	
II	15 (36.6)	10 (33.3)	
III	11 (26.8)	9 (30.0)	

Surgical performance is detailed in Table 2. Robotic procedures showed significantly higher operative times when compared to laparoscopic surgery. No significant difference was found between the two groups in terms of blood loss. More Billroth II reconstructions were performed in the RDG group even if the difference was not statistically significant. No patients required open conversion in either group. No tumor involvement of the proximal or distal margin was found in any patient in either of the two groups. A higher number of lymph nodes was retrieved and examined in the RDG group when compared with the LDG group after D2 dissection (39.1 ± 3.7 vs 30.5 ± 2.0, respectively, *P* = 0.02).

**Table 2:** Comparison of surgical performance between the laparoscopic and the robotic groups

	Laparoscopic group	Robotic group	*P* value
	*N* = 41	*N* = 30	
Type of reconstruction			NS
Billroth II	22 (53.7 %)	21 (70.0 %)	
Rou-en-Y	19 (46.3 %)	9 (30.0 %)	
Lymph node dissection			NS
D1 + α/β	4 (9.8 %)	2 (6.6 %)	
D2	37 (90.2 %)	28 (93.3 %)	
Mean operative time (min) (mean ± SEM)	262.6 ± 8.6	312.6 ± 15.7	<0.001
Blood loss (ml) (mean ± SEM)	118.7 ± 10.7	99.5 ± 7.6	NS
Conversion to open surgery	0	0	NS
Positive resection margin	0	0	NS
No. of retrieved lymph nodes after D2 dissection (mean ± SEM)	30.5 ± 2.0	39.1 ± 3.7	0.02

The incidence of postoperative complications (surgery-related and surgery-unrelated), reoperations and mortality rates were similar in the two groups (Table 3). There were two mortalities in the LDG group and one in the RDG group. The cause of the two mortalities in the LDG group included one duodenal stump leakage with peritonitis and sepsis and one case of acute myocardial infarction. The case of duodenal stump leakage occurred before the introduction of the manual reinforcement with a running suture over the duodenal stump closure. One 89-year-old female patient in the RDG group who experienced a postoperative intestinal occlusion received laparotomy but eventually died of a cerebral vascular accident.

**Table 3:** Comparison of short-term clinical outcomes between the laparoscopic and the robotic groups

	Laparoscopic group	Robotic group	*P* value
	*N* = 41	*N* = 30	
Time-to-first flatus (day) (mean ± SD)	3.0 ± 0.3	3.2 ± 0.3	NS
Time-to-first oral feeding (day) (mean ± SD)	5.4 ± 0.5	5.2 ± 0.3	NS
Surgery-related complications (total)	5 (12.1 %)	4 (13.2 %)	NS
Focal pancreatitis	1 (2.4 %)	0	
Duodenal stump leakage	2 (4.9 %)	0	
Intestinal obstruction	0	2 (6.6 %)	
Anastomotic bleeding	1 (2.4 %)	0	
Delayed gastric emptying	1 (2.4 %)	2 (6.6 %)	
Surgery-unrelated complications (total)	3 (7.2 % )	2 (6.6 %)	NS
Urinary tract infections	1 (2.4 %)	0	
Arrhythmia	1 (2.4 %)	0	
Deep venous thrombosis	0	1 (3.3 %)	
Cerebral vascular accident	0	1 (3.3 %)	
Myocardial infarction	1 (2.4 %)	0	
Reoperations	2 (4.9 %)	1 (3.3 %)	NS
Postoperative mortality	2 (4.9 %)	1 (3.3 %)	NS
Hospital length stay (day) (mean ± SD)	8.1 ± 0.5	9.5 ± 1.0	NS

No significant differences were found between the two groups in terms of time to diet, bowel function recovery or length of hospital stay (Table 3).

## Discussion

The clinical efficacy and advantages of the laparoscopic technique in the treatment of gastric cancer have now been recognized [21]. However, laparoscopic gastric surgery is still considered a technically demanding procedure. In particular, the technical threshold of performing lymph node dissection and intracorporeal suture during laparoscopic gastrectomy remains high and requires a steep learning curve [10, 11]. The robotic platform provides some technical improvements, such as improved vision, wristed instrument, tremor filtration system and motion scaling, that enable surgeons to easily perform precise lymphadenectomy and anastomoses. A number of studies have shown the feasibility and safety of robotic gastric surgery but a clear superiority of robotic surgery over laparoscopy has not yet been demonstrated [22–26]. No substantial reduction in time-to-first flatus, time-to-first oral feeding and length of hospital stay has been reported after robotic surgery when compared to laparoscopy. Our early experience in robotic gastrectomy confirms these previously published results: we did not find any significant difference in short-term clinical outcomes between patients in the robotic and those in the laparoscopic group. However, our inability to show robotic surgery to be superior to laparoscopic surgery is not surprising in light of previous studies that have compared laparoscopic with open surgery. In numerous studies, laparoscopic gastrectomy facilitated less blood loss, earlier bowel function recovery and shorter length of stay than open gastrectomy [27]. Thus, conceivably, optimal perioperative surgical outcomes may have already been achieved with laparoscopic surgery, leaving little room for improvement via robotic surgery.

One crucial step in gastric cancer surgery is lymphadenectomy since the removal of an adequate number of lymph nodes has been shown to improve the accuracy of staging and regional disease control [28]. This procedure is typically considered to be technically difficult to perform in conventional laparoscopic surgery, especially when D2 lymphadenectomy is mandatory [10, 11, 29]. This is mainly due to the use of conventional straight forceps in laparoscopic surgery that do not allow the surgeon to reach deep-seated vessels and areas such as the suprapancreatic one. Stable exposure and use of wristed instruments with the robotic system may help the surgeon to efficiently perform lymph node dissection in these delicate areas, in particular around the posterior aspect of the common hepatic artery and the splenic vessels [30]. In the present study, we found that robotic surgery can improve the quality of lymphadenectomy in distal gastric resection when compared with conventional laparoscopy. Indeed, the mean number of retrieved lymph nodes in the robotic group was significantly higher than in the laparoscopic group (39.1 vs 30.5, respectively) and, importantly, the mean values in both groups were much higher than the recommended number (i.e., 25) for adequate D2 lymphadenectomy [31]. Importantly, this number was even higher than what we found in a group of matched patients who were operated on by open distal gastrectomy between 2008 and 2012 at our institution [20].

Despite the evident technical advantages offered by the robotic system, recent meta-analyses comparing robotic and laparoscopic gastrectomy have failed to show a significant increase in the number of retrieved lymph nodes in patients operated robotically [15–17, 32]. This may be explained by the fact that the majority of the analyzed studies were carried out in the Far East where patients generally have a low BMI. Recently, Lee et al. [33] have shown that the benefits of a robotic approach were more evident in high versus normal BMI patients when performing distal gastrectomy with D2 lymphadenectomy, particularly in terms of achieving a consistent number of retrieved lymph nodes (>25). The authors concluded that robotic surgery may overcome the technical difficulties due to excessive intra-abdominal fat and thick abdominal walls during laparoscopic lymphadenectomy. Our findings seem to confirm these previously published results: the BMIs of our patients (26.0 and 27.0 kg/m^2^ in the laparoscopic and robotic group, respectively) were similar to those of high-BMI patients reported by Lee et al. (26.8 and 26.9 kg/m^2^ in the two groups, respectively), thus showing that robotic surgery may offer consistent quality of lymphadenectomy for patients with high BMI. Importantly, the present results were achieved during our very early experience in gastric robotic surgery. This suggests that surgeons with sufficient experience in laparoscopic gastrectomy can rapidly overcome the learning curve for robotic gastrectomy and high-quality surgery is achievable even after a relatively low number of cases [34]. These advantages could be more helpful in Western countries or lower volume centers, where high BMI patients are more common and where there is a lower incidence of gastric cancer, which limits the number of gastric cancer surgeries to be performed through a minimally invasive approach.

All sorts of studies that have been published about robotic gastric surgery, have reported that operative time was prolonged when compared with the laparoscopic approach and our findings are in line with these results [15–17, 32]. There are a number of possible reasons for this: first, robotic surgery is associated with an increased set-up time needed to position the robot before beginning surgery. However, docking times can be shortened after accumulation of greater experience. Secondly, the prolonged time may be due to camera motion interrupting the operative procedure and the unadapted optical system with an absence of a large general view of the operative field which prevents a safe continuous dissection and necessitates slow manipulation. However, longer operation times have never been shown to translate into increased perioperative complications and thus should not discourage surgeons from investigating the novel utility of robotic surgery.

One of the limitations of the present study was the lack of a detailed comparative analysis of cost-effectiveness between robotic and laparoscopic gastric surgery. Robotic gastric surgery undoubtedly has higher costs than laparoscopic surgery as clearly demonstrated by Park et al. [35]. The only way its use can be justified would be through improved patient survival achieved through more efficient surgery. The present study seems to show potentially relevant advantages, such as a higher number of retrieved lymph nodes, that would justify the higher costs of robotic systems. However, a multicenter, randomized study is needed to confirm this clinical benefit and evaluate whether it may effectively translate into improvement of long-term patient survival and quality of life.

## Conclusions

Within the limitation of a small-sized, non-randomized analysis, our study confirms that robot-assisted gastrectomy is a feasible and safe surgical procedure. When compared with conventional laparoscopy, robotic surgery shows evident benefits in performing lymphadenectomy with a higher number of retrieved and examined lymph nodes.

## Abbreviations

LDG: Laparoscopic distal gastrectomy
RDG: Robotic distal gastrectomy
BMI: Body mass index
AJCC: American joint committee of cancer
